# Inter-pregnancy Weight Change and Risks of Severe Birth-Asphyxia-Related Outcomes in Singleton Infants Born at Term: A Nationwide Swedish Cohort Study

**DOI:** 10.1371/journal.pmed.1002033

**Published:** 2016-06-07

**Authors:** Martina Persson, Stefan Johansson, Sven Cnattingius

**Affiliations:** 1 Clinical Epidemiology Unit, Department of Medicine–Solna, Karolinska Institutet, Stockholm, Sweden; 2 Department of Clinical Science and Education, Södersjukhuset, Karolinska Institutet, Stockholm, Sweden; University of Manchester, UNITED KINGDOM

## Abstract

**Background:**

Maternal overweight and obesity are associated with increased risks of birth-asphyxia-related outcomes, but the mechanisms are unclear. If a change of exposure (i.e., maternal body mass index [BMI]) over time influences risks, this would be consistent with a causal relationship between maternal BMI and offspring risks. Our objective was to investigate associations between changes in maternal BMI between consecutive pregnancies and risks of birth-asphyxia-related outcomes in the second offspring born at term.

**Methods and Findings:**

This study was a prospective population-based cohort study that included 526,435 second-born term (≥37 wk) infants of mothers with two consecutive live singleton term births in Sweden between January 1992 and December 2012.

We estimated associations between the difference in maternal BMI between the first and second pregnancy and risks of low Apgar score (0–6) at 5 min, neonatal seizures, and meconium aspiration in the second-born offspring. Odds ratios (ORs) were adjusted for BMI at first pregnancy, maternal height, maternal age at second delivery, smoking, education, mother´s country of birth, inter-pregnancy interval, and year of second delivery. Analyses were also stratified by BMI (<25 versus ≥25 kg/m^2^) in the first pregnancy.

Risks of low Apgar score, neonatal seizures, and meconium aspiration increased with inter-pregnancy weight gain. Compared with offspring of mothers with stable weight (BMI change of −1 to <1 kg/m^2^), the adjusted OR for a low Apgar score in the offspring of mothers with a BMI change of 4 kg/m^2^ or more was 1.33 (95% CI 1.12–1.58). The corresponding risks for neonatal seizures and meconium aspiration were 1.42 (95% CI 1.00–2.02) and 1.78 (95% CI 1.19–2.68), respectively. The increased risk of neonatal seizures related to weight gain appeared to be restricted to mothers with BMI < 25 kg/m^2^ in the first pregnancy. A study limitation was the lack of data on the effects of obstetric interventions and neonatal resuscitation efforts.

**Conclusions:**

Risks of birth-asphyxia-related outcomes increased with maternal weight gain between pregnancies. Preventing weight gain before and in between pregnancies may improve neonatal health.

## Abstract 2

Using data from the Swedish Medical Birth Register, Martina Persson and colleagues examine the associations between inter-pregnancy weight change and risks of severe birth asphyxia-related outcomes in singleton infants born at term.

## Author Summary

**Why Was This Study Done?:**

- The increasing prevalence of overweight and obesity has epidemic proportions, also among pregnant women.
- Maternal overweight and obesity increase the risks of pregnancy complications and adverse neonatal outcomes, including severe birth asphyxia disorders.
- An independent association between maternal body mass index (BMI) and birth asphyxia would be further supported if maternal weight gain between pregnancies increases the risks of birth asphyxia in the following pregnancy.

**What Did the Researchers Do and Find?:**

- Data from the nationwide Swedish Medical Birth Register were used, which included 532,858 second-born infants born at term (≥37 weeks gestation) to mothers having their first and second infants between 1992 and 2012.
- We estimated the associations between inter-pregnancy weight change and the risks of a low Apgar score (0–6) at 5 min (rate 5.4/1,000), neonatal seizures (rate 1.2/1,000), and meconium aspiration (rate 0.7/1,000).
- The risks of a low Apgar score consistently increased with maternal weight gain. Compared with mothers with stable weight (−1 to <1 kg/m^2^ change in BMI between pregnancies), infants born to mothers who gained 4 kg/m^2^ or more between pregnancies had a 33% to 78% increased risk of low Apgar score, neonatal seizures, and meconium aspiration.
- A study limitation is that we could not investigate the potential impact of obstetric and neonatal interventions.

**What Do These Findings Mean?:**

- The risk of birth asphyxia increases with maternal weight gain between pregnancies. Consequently, preventing weight gain between pregnancies could reduce the risk of birth asphyxia and improve infant health.

## Introduction

Maternal overweight and obesity during pregnancy increase the risks of severe maternal and infant complications [1–4]. In Sweden, the proportion of women with overweight and obesity (body mass index [BMI] ≥ 25 kg/m^2^) in early pregnancy increased from 26% in 1992 to 38% in 2010 [5]. In the US, 58% of women between 20 and 39 y of age were overweight or obese in 2011–2012 [6]. As recently stated by WHO, the prevalence of maternal obesity must be reduced in order to improve maternal, fetal, and neonatal health [7].

Maternal overweight and obesity increase the risks of severe neonatal complications, including major malformations, preterm birth, neonatal morbidities, and low Apgar score (0–6) [3,8–11]. In term non-malformed infants, low Apgar score is commonly caused by birth asphyxia [12].

We have previously demonstrated a linear relationship between maternal BMI in early pregnancy and the risks of low Apgar scores at 5 and 10 min and birth-asphyxia-related neonatal morbidity in infants born at term [8]. Furthermore, we have found that the risk of asphyxia-related infant mortality in term infants increases with the degree of maternal obesity [5]. Risks being influenced by a change of exposure (i.e., maternal BMI) over time would be consistent with a causal relationship between maternal BMI and adverse outcomes of the offspring.

In this nationwide Swedish cohort study, we examined whether changes in maternal BMI between first and second pregnancies influenced the risks of birth-asphyxia-related outcomes in the second-born offspring, including low Apgar score (0–6) at 5 min, neonatal seizures, and meconium aspiration.

## Methods

### Study Design and Population

Ethics approval for this study was obtained from the Research Ethics Committee at Karolinska Institutet in Stockholm, Sweden (number 2012/4:9). Informed consent was not required as all data were anonymous.

Between January 1992 and December 2012, the nationwide Swedish Medical Birth Register included 533,535 mothers with first and second live singleton term births (≥37 completed weeks). We excluded 165 mothers with no information on inter-pregnancy interval and 512 mothers with no information on mothers’ country of birth. In analyses of low Apgar score in the second infant, we excluded another 6,423 mothers (1.2%) where either the first- or the second-born infant had missing information on Apgar score at 1 or 5 min.

The Swedish Medical Birth Register started in 1973 and contains prospectively collected data on more than 98% of all births in Sweden. The quality of data is considered high [13]. Information on sociodemographic factors, maternal and infant anthropometry, and Apgar scores are collected on standardized forms used in antenatal, obstetric, and neonatal care in Sweden. Maternal and neonatal diagnoses, including pregnancy complications and neonatal morbidities, are classified by physicians according the Swedish version of the International Classification of Diseases (ICD). The ninth version (ICD-9) was used between 1992 and 1996, and the tenth version (ICD-10) thereafter. The standardized forms are forwarded to the Swedish Medical Birth Register when the mother and infant are discharged from hospital. Information on the mother’s country of birth and level of education were obtained from the Swedish Register of Total Population and the Swedish Register of Education, respectively. Individual cross-linkage of registries was possible using the personal identification number [14], a person-unique identifier assigned to all Swedish citizens at birth or at naturalization.

### Exposures

Maternal BMI (kg/m^2^) was calculated for each of the two consecutive pregnancies. At the first antenatal visit, which occurs in the first trimester in 90% of pregnancies [13], the woman´s weight is measured wearing light indoor clothes and barefoot, and data on self-reported height are recorded. Inter-pregnancy change in BMI was calculated as the difference in BMI between the second and first pregnancies. We categorized the inter-pregnancy BMI change as <−2 kg/m^2^ (i.e., BMI loss greater than 2 kg/m^2^), −2 to <−1 kg/m^2^, −1 to <1 kg/m^2^ (stable weight), 1 to <2 kg/m^2^, 2 to <4, and ≥4 kg/m^2^. A 1-kg/m^2^ change in BMI corresponds to change of 2.8 kg in a woman of average height (167 cm). Based on BMI in the first pregnancy, mothers were categorized as underweight (BMI < 18.5 kg/m^2^), normal weight (18.5–24.9 kg/m^2^), overweight (25–29.9 kg/m^2^), obese grade I (30–34.9 kg/m^2^), and obese grade II-III (≥35 kg/m^2^) [15].

The inter-pregnancy interval was calculated as the time difference between the date of birth of the first infant and estimated date of conception of the second infant (i.e., date of birth of the second infant minus gestational age + 14 d). Gestational age was estimated primarily based on early second trimester ultrasound, which is offered to all pregnant women and which 95% of women accept [16]. When dating from an ultrasonic scan was not available, gestational age was estimated using information on date of last menstrual period. Obesity-related disorders were identified in the Swedish Medical Birth Register based on ICD codes: preeclampsia—ICD-9 codes 642E–642G, ICD-10 codes O14–O15; chronic hypertension—ICD-9 codes 401–405, 642C, 642M, ICD-10 codes O10–O11; gestational diabetes—ICD-9 code 648W, ICD-10 code O244; pregestational diabetes—ICD-9 code 250, ICD-10 codes E10–E14, O241–O243. Covariates were categorized according to Table 1.

**Table 1:** Maternal characteristics and rates of low Apgar scores (0–6), meconium aspiration, and neonatal seizures: live-born singleton second term infants of women in Sweden 1992–2012.

Category	Characteristic	Number of Births	Outcome Rate/1,000		
			Apgar Score 0–6 at 5 min (*n =* 2,824)	Neonatal Seizures (*n =* 658)	Meconium Aspiration (*n =* 372)
**Total**		532,858	5.36	1.23	0.70
**Maternal factors**	**BMI at first pregnancy (kg/m** ^**2**^ **)**				
	<18.5	14,467	3.50	1.04	0.69
	18.5–24.9	326,207	4.66	1.04	0.60
	25–29.9	95,773	6.45	1.59	0.75
	30–34.9	24,233	9.38	1.86	1.16
	≥35	8,127	11.20	2.70	1.97
	Data missing	64,051	5.50	1.30	0.78
	**Maternal age at second delivery (years)**				
	≤24	58,932	4.09	1.04	0.68
	25–29	173,609	4.67	1.08	0.62
	30–34	207,307	5.46	1.29	0.67
	≥35	93,010	7.23	1.53	0.94
	**Maternal height (cm)**				
	<155	13,695	8.81	1.53	0.58
	155–164.9	180,958	6.58	1.45	0.85
	165–174.9	278,325	4.68	1.16	0.61
	≥175	52,782	3.80	0.85	0.64
	Data missing	7,098	6.06	0.84	0.70
	**Smoking in second pregnancy**				
	No	469,399	5.29	1.22	0.67
	Yes	41,048	5.95	1.46	0.95
	Data missing	22,411	5.92	1.20	0.80
	**Inter-pregnancy interval (years)**				
	<1	94,653	4.56	1.09	0.61
	1 to <3	318,314	5.22	1.17	0.65
	3 to <5	78,566	5.82	1.37	0.75
	≥5	41,325	7.49	1.81	1.19
	**Education (years)**				
	≤11	122,582	5.88	1.42	0.96
	12–14	218,822	5.42	1.24	0.64
	≥15	186,945	4.94	1.10	0.58
	Data missing	4,509	6.30	1.55	1.11
	**Mother’s country of birth**				
	Nordic	460,920	5.35	1.26	0.67
	Non-Nordic	71,938	5.44	1.10	0.90
	**Chronic hypertension**				
	No	530,249	5.34	1.23	0.70
	Yes	2,609	10.10	3.07	1.15
	**Preeclampsia**				
	No	526,702	5.29	1.21	0.70
	Yes	6,165	11.32	3.57	0.49
	**Diabetes**				
	No	526,995	5.29	1.21	0.69
	Gestational diabetes	4,079	11.67	2.45	1.23
	Pregestational diabetes	1,784	11.97	5.04	1.68
**Offspring in second pregnancy**	**Birth weight (grams)**				
	<2,500	2,809	16.99	3.20	1.78
	2,500–2,999	32,346	6.47	1.79	0.74
	3,000–3,999	364,216	4.42	1.01	0.57
	4,000–4,499	105,070	5.91	1.24	0.82
	≥4,500	27,232	10.63	2.57	1.40
	Data missing	1,185	91.20	18.56	10.13
	**Gestational age (weeks)**				
	37–38	96,571	6.09	1.30	0.27
	39–40	304,114	4.45	1.10	0.57
	41	99,113	6.44	1.35	1.22
	≥42	33,060	8.40	1.94	1.54
	**Birth weight for gestational age (percentile)**				
	<3	3,611	16.10	3.05	2.49
	3 to <10	15,247	7.51	1.77	1.44
	10 to 90	430,198	4.64	1.05	0.61
	90 to 97	57,952	6.05	1.48	0.69
	≥97	24,514	10.42	2.37	1.02
	Data missing	1,336	83.50	17.22	8.98

Rates for low Apgar score are based on offspring with data on Apgar score at 1 and 5 min, *n =* 526,435.

### Outcomes

We estimated the risks of severe asphyxia-related outcomes, including low Apgar score, meconium aspiration, and neonatal seizures, at the second birth by change in BMI from the first to the second pregnancy. A low Apgar score was defined as a score between 0 and 6 points at 5 min after birth. A diagnosis of neonatal seizures was based on ICD-9 code 779.0 or ICD-10 code P90, and a diagnosis of meconium aspiration was based on ICD-9 code 770.1 or ICD-10 code P24.0.

### Statistical Analyses

Rates of low Apgar score, meconium aspiration, and neonatal seizures were calculated as the number of infants with these outcomes per 1,000 births. Logistic regression analyses were used to calculate odds ratios (ORs) with 95% confidence intervals for all outcomes. Mothers whose first infant had a low Apgar score, meconium aspiration, or neonatal seizures were excluded from the analysis of the respective outcome in the second pregnancy to avoid bias in case of a tendency to repeat adverse pregnancy outcomes. Inter-pregnancy weight change was categorized as presented above in the regression model, but also treated as a continuous variable. Multivariate models were restricted to second births with complete data on inter-pregnancy weight change and covariates. We adjusted for maternal BMI in the first pregnancy, maternal height, maternal age at second delivery, smoking habits in the second pregnancy, inter-pregnancy interval, mother´s education, mother’s country of birth, and year of second birth, categorized according to Table 1. Given the long study period, spanning over 21 y, we categorized year of second birth (not shown in Table 1) into intervals as 1992–1996, 1997–2001, 2002–2006, and 2007–2012.

In sensitivity analyses (S1 Table), mothers with obesity-related disorders (chronic hypertension, preeclampsia, or any type of diabetes) were excluded from the regression analyses. A sensitivity analysis was also performed to explore whether pregnancies of mothers with missing data on inter-pregnancy weight change differed from those with information on inter-pregnancy weight change (S2 Table). To explore any effect modification by first pregnancy BMI on the association between exposure and outcomes, analyses were also stratified by maternal BMI in the first pregnancy (BMI < 25 or BMI ≥ 25 kg/m^2^). Interaction terms were introduced in the multivariate models, and a *p*-value of less than 0.05 for the interaction term was considered statistically significant.

## Results

The total number of infants with a low Apgar score (0–6 points) at 5 min was 2,824 (rate 4.08/1,000). Corresponding numbers (rates) for neonatal seizures and meconium aspiration were 658 (1.23/1,000) and 372 (0.70/1,000), respectively (Table 1). Rates of all birth-asphyxia-related outcomes increased with maternal BMI in the first pregnancy, inter-pregnancy interval, smoking, chronic hypertension, preeclampsia, any type of diabetes, and generally also with maternal age at second delivery (Table 1). Maternal education was inversely correlated with all outcomes. There was a U-shaped relationship between birth weight and gestational age and rates of low Apgar score and neonatal seizures, while the rate of meconium aspiration increased with gestational age. Rates of low Apgar score, neonatal seizures, and meconium aspiration in second-born offspring were essentially similar in offspring of mothers with missing data on BMI in the first pregnancy and the total population.

Rates of low Apgar score, neonatal seizures, and meconium aspiration increased with inter-pregnancy weight gain (Table 2). Compared with mothers with stable weight, the risk of a low Apgar score was 26% increased in offspring of mothers who gained 2 to <4 kg/m^2^ and 33% increased in offspring of mothers who gained ≥4 kg/m^2^. Risk of neonatal seizures was increased by more than 40% in offspring of mothers who gained 2 to <4 and ≥4 kg/m^2^. The risk of meconium aspiration was 78% higher in offspring of mothers who gained ≥4 kg/m^2^ compared with offspring of mothers with stable weight.

**Table 2:** Maternal inter-pregnancy weight change and risks of low Apgar score (0–6) at 5 min, neonatal seizures, and meconium aspiration: live singleton second term infants of women in Sweden 1992–2012.

Outcome	Inter-pregnancy Weight Change (kg/m^2^)	Number with Outcome	Rate/1,000	OR (95% CI)	
				Crude^a^	Adjusted^b^
**Low Apgar score (0–6) at 5 min**	<−2	96	4.99	1.10 (0.89–1.36)	0.84 (0.67–1.04)
	−2 to <−1	155	4.34	0.95 (0.80–1.13)	0.90 (0.76–1.07)
	−1 to <1	878	4.54	1.00	1.00
	1 to <2	458	5.41	1.19 (1.06–1.33)	1.14 (1.02–1.28)
	2 to <4	407	6.36	1.40 (1.25–1.58)	1.26 (1.11–1.42)
	≥4	178	7.54	1.66 (1.42–1.96)	1.33 (1.12–1.58)
	Data missing	564	5.60		
	Per 1-kg/m^2^ change in BMI			1.08 (1.03–1.14)	1.07 (1.04–1.09)
**Neonatal seizures**	<−2	30	1.53	1.48 (1.01–2.18)	1.18 (0.79–1.76)
	−2 to <−1	39	1.07	1.04 (0.74–1.43)	1.00 (0.71–1.42)
	−1 to <1	203	1.03	1.00	1.00
	1 to <2	105	1.22	1.18 (0.93–1.49)	1.13 (0.89–1.44)
	2 to <4	108	1.65	1.61 (1.27–2.03)	1.46 (1.14–1.86)
	≥4	44	1.82	1.77 (1.28–2.45)	1.42 (1.00–2.02)
	Data missing	120	1.17		
	Per 1-kg/m^2^ change in BMI			1.00 (0.90–1.12)	1.05 (1.01–1.09)
**Meconium aspiration**	<−2	10	0.51	0.86 (0.45–1.64)	0.53 (0.25–1.10)
	−2 to <−1	19	0.52	0.88 (0.54–1.42)	0.84 (0.52–1.37)
	−1 to <1	117	0.59	1.00	1.00
	1 to <2	51	0.59	0.99 (0.72–1.38)	0.95 (0.68–1.32)
	2 to <4	54	0.83	1.39 (1.01–1.92)	1.14 (0.81–1.60)
	≥4	38	1.58	2.66 (1.84–3.83)	1.78 (1.19–2.68)
	Data missing	78	0.76		
	Per 1-kg/m^2^ change in BMI			1.15 (1.02–1.29)	1.11 (1.06–1.17)

Women whose first infant had a low Apgar score (0–6) at 5 min (*n =* 5,852), neonatal seizures (*n =* 1,325), or meconium aspiration (*n =* 1,380) are excluded from respective analyses.

^a^Crude analyses are based on the following number of second births: Apgar score, *n =* 420,429 infants, of whom 2,172 had a low Apgar score (0–6) at 5 min; neonatal seizures, *n =* 428,945, of whom 529 had neonatal seizures; meconium aspiration, *n =* 428,908, of whom 289 had meconium aspiration.

^b^Adjusted for BMI in first pregnancy, maternal height, smoking in second pregnancy, maternal age at second birth, inter-pregnancy interval, mother’s education, mother’s country of birth, and year of second birth. Adjusted analyses are based on second births with complete information on maternal covariates (Apgar score: 412,938 infants, of whom 2,128 had Apgar score 0–6 at 5 min; neonatal seizures: 421,250 infants, of whom 518 had neonatal seizures; meconium aspiration: 421,206 infants, of whom 280 had meconium aspiration).

We investigated whether the effect of weight change on asphyxia-related outcomes differed between offspring of mothers who were underweight/normal weight and overweight/obese in the first pregnancy (BMI <25 and ≥25 kg/m^2^, respectively) (Table 3). In underweight/normal weight mothers in the first pregnancy, the risk of low Apgar score increased with weight gain, while the corresponding association was less evident in infants of mothers who were overweight/obese in the first pregnancy. However, the test for an interaction between maternal BMI and low Apgar was not significant (*p* = 0.12).

**Table 3:** Maternal inter-pregnancy weight change and risk of low Apgar score (0–6) at 5 min, neonatal seizures, and meconium aspiration, stratified by maternal BMI in first pregnancy: live singleton second term infants of women in Sweden 1992–2012.

Outcome	Inter-pregnancy Weight Change (kg/m^2^)	Maternal BMI < 25 kg/m^2^		Maternal BMI ≥ 25 kg/m^2^		Interaction *p*-Value
		Number with Outcome (Rate/1,000)	OR (95% CI)	Number with Outcome (Rate/1,000)	OR (95% CI)	
**Low Apgar score (0–6)**	Total	1,361 (4.45)		811 (7.06)		0.12
	<−2	24 (3.47)	0.82 (0.54–1.26)	72 (5.84)	0.83 (0.63–1.09)	
	−2 to <−1	81 (3.34)	0.83 (0.66–1.05)	74 (6.44)	0.98 (0.75–1.27)	
	−1 to <1	633 (4.08)	1.00	245 (6.43)	1.00	
	1 to <2	314 (4.88)	1.16 (1.01–1.33)	144 (7.10)	1.10 (0.89–1.35)	
	2 to <4	225 (5.33)	1.24 (1.06–1.46)	182 (8.37)	1.26 (1.04–1.53)	
	≥4	84 (6.63)	1.50 (1.18–1.92)	94 (8.59)	1.20 (0.94–1.55)	
**Neonatal seizures**	Total	322 (1.10)		196 (1.70)		0.004
	<−2	5 (0.71)	0.65 (0.24–1.77)	25 (1.98)	1.25 (0.78–2.01)	
	−2 to <−1	19 (0.77)	0.87 (0.54–1.41)	20 (1.69)	1.12 (0.67–1.86)	
	−1 to <1	142 (0.90)	1.00	61 (1.57)	1.00	
	1 to <2	73 (1.11)	1.21 (0.91–1.61)	32 (1.54)	1.00 (0.65–1.54)	
	2 to <4	65 (1.51)	1.65 (1.22–2.24)	43 (1.93)	1.21 (0.81–1.81)	
	≥4	24 (1.85)	2.04 (1.30–3.22)	20 (1.79)	0.99 (0.57–1.70)	
**Meconium aspiration**	Total	181 (0.60)		99 (0.90)		0.77
	<−2	2 (0.28)	0.25 (0.03–1.78)	8 (0.63)	0.65 (0.28–1.49)	
	−2 to <−1	11 (0.44)	0.81 (0.43–1.52)	8 (0.68)	0.90 (0.41–1.96)	
	−1 to <1	88 (0.56)	1.00	29 (0.74)	1.00	
	1 to <2	28 (0.43)	0.74 (0.48–1.13)	23 (1.10)	1.46 (0.85–2.53)	
	2 to <4	36 (0.84)	1.27 (0.84–1.92)	18 (0.81)	0.96 (0.52–1.75)	
	≥4	23 (1.77)	2.22 (1.32–3.73)	15 (1.34)	1.46 (0.76–2.80)	

ORs were adjusted for BMI in first pregnancy, smoking in second pregnancy, maternal age at second birth, inter-pregnancy interval, mother’s education, mother’s country of birth, and year of second birth. Among mothers with BMI < 25 kg/m^2^, the adjusted analyses of low Apgar score include 300,002 second births with complete information on covariates; 1,334 infants had a low Apgar score. Among mothers with BMI ≥ 25 kg/m^2^, the corresponding numbers are 112,936 second births and 794 infants with low Apgar score. Among mothers with BMI < 25 kg/m^2^, the adjusted analyses of neonatal seizures include 305,670 second births with complete covariate information; 322 infant had neonatal seizures. Among mothers with BMI ≥ 25 kg/m^2^, the corresponding numbers are 115,580 second births and 196 infants with neonatal seizures. Among mothers with BMI < 25 kg/m^2^, the adjusted analyses of meconium aspiration include 305,653 second births with complete covariate information; 181 infants had meconium aspiration. Among mothers with BMI ≥ 25 kg/m^2^, the corresponding numbers are 115,553 second births and 99 infants with meconium aspiration.

The risk of neonatal seizures increased with weight gain in offspring of mothers who were underweight/normal weight in the first pregnancy, but not in offspring of mothers who were overweight/obese in the first pregnancy (test for interaction; *p* = 0.004). In underweight/normal weight mothers, offspring of mothers with a weight gain of ≥4 kg/m^2^ had a doubled risk of neonatal seizures compared with offspring of mothers with stable BMI.

Stratifying the analyses by maternal BMI in the first pregnancy (<25 and ≥25 kg/m^2^) demonstrated a more than doubled risk of meconium aspiration in offspring of underweight/normal weight mothers who gained ≥4 kg/m^2^. In contrast, in overweight/obese mothers, weight gain was not associated with increased risk of meconium aspiration in offspring. However, the test for interaction was not significant (*p* = 0.77).

In order to investigate whether associations between maternal weight gain and asphyxia-related outcomes were influenced by obesity-related disorders, we repeated the analyses after excluding offspring of mothers with chronic hypertension, preeclampsia, or any type of diabetes. Restricting the analyses to offspring of mothers without obesity-related disorders did not change the risks of low Apgar score, neonatal seizures, or meconium aspiration (S1 Table).

We also investigated weight gain and the risks of birth-asphyxia-related outcomes in the offspring of mothers who were underweight/normal weight (BMI below 25 kg/m^2^) in the second pregnancy (S2 Table). Compared with the offspring of mothers with stable weight, the risks of low Apgar score and neonatal seizures were more than doubled in offspring of mothers who were normal weight in the second pregnancy but who had gained ≥4 kg/m^2^ between pregnancies.

Finally, we investigated whether mothers with missing information on inter-pregnancy weight gain, and hence not included in the analyses, differed from mothers with information on weight gain with respect to other maternal characteristics or birth outcomes. The proportions of women who were overweight, obesity grade I, and obesity grade II-III in the first pregnancy were 20.5%, 5.2%, and 1.7%, respectively, for mothers with known weight change and 19.3%, 4.9%, and 1.9%, respectively, for mothers with unknown weight change (due to missing data on second pregnancy BMI) (S3 Table). Also, the distributions of maternal covariates (as listed in Table 1) were comparable between mothers with missing data on inter-pregnancy weight change and mothers with an inter-pregnancy weight change of −1 and <1 kg/m^2^ (the reference group) or an inter-pregnancy weight gain of 1 to <2 kg/m^2^. The proportions of mothers with obesity-related diseases and rates of birth-asphyxia-related outcomes were similar in mothers with and without data on inter-pregnancy weight change (S3 Table).The proportions of women who were overweight, obesity grade I, and obesity grade II-III in the second pregnancy were 25.3%, 7.6%, and 1.4%, respectively, for mothers with information on inter-pregnancy weight change and 24.2%, 7.1%, and 1.4%, respectively, for mothers with unknown weight change (due to missing data on first pregnancy BMI).

## Discussion

### Principal Findings

In this population-based cohort study we found that the risks of severe birth-asphyxia-related outcomes in the second offspring born at term increased with inter-pregnancy maternal weight gain. The risk increases were primarily found in offspring of mothers with BMI < 25 kg/m^2^ in the first pregnancy. The observed increments in risks of low Apgar score, neonatal seizures, and meconium aspiration remained essentially the same after exclusion of offspring of mothers with obesity-related diseases.

### Findings in Comparison with Other Studies

Data from epidemiological studies support that maternal BMI and changes in maternal BMI influence the risks of maternal complications, preterm delivery, and infant mortality [8–11,17–23], We have previously reported that the risks of severe birth-asphyxia-related complications increase with maternal overweight and obesity [8]. To our knowledge, this is the first study to assess whether these risks are influenced by changes of exposure (i.e., change in weight) over time.

The pathophysiology underlying the associations between maternal overweight/obesity and birth-asphyxia-related outcomes in offspring is likely to be complex and multifactorial. Obesity in pregnant women is accompanied by inflammation in maternal and placental tissues, impaired microvascular function, oxidative stress, and marked insulin resistance [24,25], changes that may contribute to the increased risk of birth asphyxia and other complications. It has also been proposed that an altered gut microbiota in obese women adversely influences maternal metabolism, which in turn may affect fetal health [26].

Fetal macrosomia is the most prevalent complication in pregnancies with maternal obesity [4]. There is a linear association between maternal BMI and fetal macrosomia and between maternal BMI and measures of hyperinsulinemia in cord blood [27], independent of maternal glucose values. Results from both experimental and clinical studies strongly suggest that fetal hyperinsulinemia is a risk factor for fetal hypoxia [28–31]. Thus, it is possible that fetal hyperinsulinemia is of pathophysiological importance for the increased risk of birth asphyxia in pregnancies with maternal obesity. Fetal macrosomia also increases the risks of traumatic delivery and shoulder dystocia, which increase the risk of birth asphyxia [32]. Obesity-related disorders, including chronic hypertension, preeclampsia, and diabetic diseases, are associated with increased risks of fetal hypoxia and low Apgar score [33,34]. However, excluding offspring of mothers with obesity-related diseases did not substantially change the weight-gain-related risks of asphyxia-related outcomes in our study.

Compared with offspring of normal weight mothers, offspring of overweight and obese mothers are at increased risks of birth-asphyxia-related neonatal outcomes [8]. However, increments in risks associated with inter-pregnancy weight gain were primarily restricted to offspring of mothers with BMI < 25 kg/m^2^ in the first pregnancy. The same pattern has also been demonstrated for complications during pregnancy [17]. Interestingly, in offspring of mothers with BMI < 25 kg/m^2^ in the second pregnancy, risks of low Apgar score and neonatal seizures were more than doubled in offspring of mothers who gained ≥4 kg/m^2^ between pregnancies (S2 Table). One could speculate that the less pronounced effect of weight gain in women with established overweight/obesity may reflect a metabolic adaption over time to an increased fat mass. Furthermore, women with overweight or obesity in early pregnancy accumulate less fat in pregnancy than underweight/normal weight women [25]. Thus, the same absolute increase in weight would reflect a larger relative increase in fat mass in women of underweight/normal weight compared with women who already were overweight. It is also possible that the distribution of added fat mass between pregnancies differs between lean women and overweight women, with a relatively larger increment in visceral fat in lean than in overweight women. However, rates of birth-asphyxia-related outcomes also increased with weight gain in offspring among women who were overweight or obese in the first pregnancy. Thus, the statistical power to detect risk increments could have been insufficient.

### Strengths and Limitations of the Present Study

The primary strengths of our study are the population-based design, including a large number of births, and the prospectively recorded data on exposures and outcomes. The large cohort enabled us to investigate the impact of a range of inter-pregnancy weight change categories on the risks of neonatal outcomes related to birth asphyxia. The prospectively collected data limited the risks of selection and information bias. We were also able to analyze these risks stratified by maternal BMI in the first pregnancy and to adjust for several potential confounders. We used maternal BMI as a proxy for maternal fat mass. This assumption is justified as there is a strong correlation (*r* ^2^ = 0.84) between BMI and fat mass in early pregnancy [35].

Some limitations of the present study should be noted. In the present study, inter-pregnancy weight change was calculated as the difference in early pregnancy BMI between the two first consecutive pregnancies. We did not have information on gestational weight gain and do not know when the weight gain occurred. The distribution of fat may differ if weight is gained during or after pregnancy. Longitudinal studies demonstrate that women with high gestational weight gain are likely to retain this weight into subsequent pregnancies [36] and that high weight gain in the first pregnancy is an important risk factor for future overweight and obesity [37].

In spite of having data on many key confounders, we cannot rule out the possibility of residual confounding by unmeasured maternal factors driving the relationship between inter-pregnancy weight change and risk of birth asphyxia. For example, women who gain weight between pregnancies may also have a less healthy life style in other aspects than women with stable weight. We also lacked specific information on obstetric interventions and neonatal resuscitation efforts. Therefore, the impact of these factors on the risk of birth asphyxia could not be investigated.

We studied neonatal conditions related to birth asphyxia. Severe birth asphyxia is commonly defined as an Apgar score of 0–3 at 5 min in combination with cord blood acidosis and neurological symptoms like neonatal seizures [38]. However, in epidemiological studies, a frequently used definition of birth asphyxia is an Apgar score of 0–6 at 5 min [39,40]. The validity of this definition is supported by the fact that an infant with an Apgar score of 4–6 at 5 min has a 45 times higher risk of neonatal death and a 31 times higher risk of cerebral palsy compared to infants with an Apgar score of 7–10 at 5 min [41,42]. We studied only infants born at term, as preterm birth itself is a common reason for low Apgar score [43]. Maternal BMI in early pregnancy also influences risk of preterm birth [10]. In addition, the possibility of selection bias should be considered given that the analyses were restricted to offspring of mothers with two children.

Information on inter-pregnancy weight change was missing in 19% of the study population. If pregnancies with information on inter-pregnancy weight change differ from pregnancies without this information, results could be biased. However, the distributions of maternal covariates, overweight, and obesity were similar in the first and second pregnancies of women with and without information on inter-pregnancy weight change, and rates of asphyxia-related outcomes were also similar.

### Summary

Given the high prevalence of maternal overweight and the possible long-term consequences of birth asphyxia, our results have substantial public health relevance, as even modest weight increases in normal weight women may impact offspring outcomes on a population level. However, our finding that inter-pregnancy weight gain influences the risks of birth-asphyxia-related outcomes should be confirmed in other populations. Encouraging women to normalize BMI before pregnancy and to avoid weight gain between pregnancies is likely to be an important measure to improve infant health.

## Supporting Information

S1 STROBE Statement Checklist of items that should be included in reports of observational studies. (DOC)

S1 Table Maternal inter-pregnancy weight change and risk of low Apgar score (0–6) at 5 min, meconium aspiration and neonatal seizures: live singleton term infants of mothers without obesity-related pregnancy complications in second pregnancy. (DOCX)

S2 Table Maternal inter-pregnancy weight change and risk of low Apgar score, neonatal seizures, and meconium aspiration in second offspring: mothers with BMI 18.5–24.9 kg/m^2^ in second pregnancies with live singleton term infants at second birth, Sweden 1992–2012. (DOCX)

S3 Table Characteristics of women with and without data on inter-pregnancy weight change. (DOCX)

S1 Text Analysis plan. (DOCX)

## Abbreviations

BMI: body mass index
ICD: International Classification of Diseases
OR: odds ratio
