# Supplementary material for: Inter-pregnancy Weight Change and Risks of Severe Birth-Asphyxia-Related Outcomes in Singleton Infants Born at Term: A Nationwide Swedish Cohort Study
Source: PLoS Med. 2016 Jun 7;13(6):e1002033. doi: 10.1371/journal.pmed.1002033 (PMC4896455; doi:10.1371/journal.pmed.1002033)
Supplement: S1 Table — (DOCX) [file pmed.1002033.s002.docx]

**S1 Table . Maternal interpregnancy weight change and risk of low Apgar score (0-6) at five minutes, meconium aspiration and neonatal seizures. Live singleton term infants of mothers without obesity-related pregnancy complications in second pregnancy.**

|  |  | **Low (0-6) Apgar score at 5 minutes** | | | | |
| --- | --- | --- | --- | --- | --- | --- |
|  |  |  |  | OR (95% CI) | | |
| Interpregnancy weight change (BMI units) | Total No. | No. | Rate/1000 | Crude^a^ | Adjusted^b^ | |
| <-2 | 18,685 | 94 | 5.03 | 1.13 (0.91-1.40) | 0.87 (0.70-1.09) | |
| -2 to <-1 | 34,961 | 145 | 4.15 | 0.93 (0.78-1.11) | 0.88 (0.73-1.05) | |
| -1 to <1 | 189,349 | 845 | 4.46 | 1.00 | 1.00 | |
| 1 to <2 | 82,418 | 438 | 5.31 | 1.19 (1.06-1.34) | 1.14 (1.02-1.29) | |
| 2 to <4 | 61,686 | 380 | 6.16 | 1.38 (1.22-1.56) | 1.24 (1.10-1.41) | |
| ≥4 | 22,196 | 155 | 6.98 | 1.57 (1.32-1.86) | | 1.28 (1.06-1.53) |
| Data missing | 100,122 | 524 | 5.5 |  | |  |
|  |  | **Neonatal seizures** | | | | |
|  |  |  |  | OR (95% CI) | | |
| Interpregnancy weight change (BMI units) | Total No. | No. | Rate/1000 | Crude^a^ | | Adjusted^b^ |
| <-2 | 19,082 | 28 | 1.47 | 1.50 (1.01-2.23) | | 1.23 (0.81-1.87) |
| -2 to <-1 | 35,681 | 37 | 1.04 | 1.06 (0.74-1.51) | | 1.03 (0.72-1.47) |
| -1 to <1 | 193,029 | 189 | 0.98 | 1.00 | | 1.00 |
| 1 to <2 | 84,051 | 102 | 1.21 | 1.24 (0.97-1.58) | | 1.20 (0.94-1.53) |
| 2 to <4 | 63,000 | 99 | 1.57 | 1.61 (1.26-2.05) | | 1.48 (1.15-1.91) |
| ≥4 | 22,678 | 38 | 1.68 | 1.71 (1.21-2.43) | | 1.41 (0.97-2.05) |
|  |  | **Meconium aspiration** | | | | |
|  |  |  |  | OR (95% CI) | | |
| Interpregnancy weight change (BMI units) | Total No. | No. | Rate/1000 | Crude^a^ | | Adjusted^b^ |
| <-2 | 19,070 | 10 | 0.52 | 0.87 (0.46-1.67) | | 0.52 (0.25-1.09) |
| -2 to <-1 | 35,691 | 17 | 0.48 | 0.79 (0.48-1.32) | | 0.75 (0.45-1.26) |
| -1 to <1 | 192,990 | 116 | 0.60 | 1.00 | | 1.00 |
| 1 to <2 | 84,066 | 47 | 0.56 | 0.93 (0.66-1.31) | | 0.88 (0.63-1.24) |
| 2 to <4 | 62,989 | 54 | 0.86 | 1.43 (1.03-1.97) | | 1.15 (0.82-1.62) |
| ≥4 | 22,660 | 37 | 1.63 | 2.72 (1.88-3.94) | | 1.79 (1.18-2.70) |
| Data missing | 416,535 |  |  |  | |  |
|  |  |  |  |  | |  |

^a^Crude odds ratios are based on 409,295second births with information on interpregnancy weight change, of whom 2,057 had low (0-6) Apgar score at 5 minutes.

^b^Adjusted for BMI in first pregnancy, smoking in 2^nd^ pregnancy, maternal age at second birth, interpregnancy interval, mother’s education and country of birth, and year of 2^nd^ birth. Adjusted analyses for Apgar score 0-6 at 5 minutes are based on 400,012 second births with complete information on co-variates. Number of births with Apgar score 0-6 at 5 minutes were 2,014. Adjusted analyses for neonatal seizures and meconium aspiration syndrome are based on 409,081 second births with complete information on co-variates. Number of infants with neonatal seizures were 478. Number of infants with meconium aspiration were 280.
