# Supplementary material for: Inter-pregnancy Weight Change and Risks of Severe Birth-Asphyxia-Related Outcomes in Singleton Infants Born at Term: A Nationwide Swedish Cohort Study
Source: PLoS Med. 2016 Jun 7;13(6):e1002033. doi: 10.1371/journal.pmed.1002033 (PMC4896455; doi:10.1371/journal.pmed.1002033)
Supplement: S2 Table — (DOCX) [file pmed.1002033.s003.docx]

**S2 Table. Maternal interpregnancy weight change and risk of low Apgar score, neonatal seizures and meconium aspiration syndrome in second offspring. Mothers with BMI 18.5-24.9 in second pregnancies with live singleton term infants at second birth, Sweden 1992-2012.**

|  |  | **Apgar 0-6** at 5 min | | | |
| --- | --- | --- | --- | --- | --- |
|  |  |  |  | OR (95% CI) | |
| Weight change (BMI units) | Total | No. | Rate/1000 | Crude^a^ | Adjusted^b^ |
| <-2 | 12,210 | 44 | 3.60 | 0.88 (0.65-1.20) | 0.81 (0.57-1.17) |
| -2 to <-1 | 26,549 | 97 | 3.35 | 0.89 (0.72-1.11) | 0.88 (0.70-1.10) |
| -1 to <1 | 147,432 | 603 | 4.09 | 1.00 | 1.00 |
| 1 to <2 | 51,973 | 238 | 4.58 | 1.12 (0.96-1.30) | 1.10 (0.94-1.28) |
| 2 to <4 | 22,799 | 102 | 4.47 | 1.09 (0.89-1.35) | 1.07 (0.86-1.33) |
| ≥4 | 2,051 | 18 | 8.78 | 2.16 (1.35-3.46) | 2.17 (1.31-3.59) |
|  |  | **Neonatal seizures** | | | |
|  |  |  |  | OR (95% CI) | |
| Weight change (BMI units) | Total | No. | Rate/1000 | Crude^a^ | Adjusted^b^ |
| <-2 | 12,452 | 14 | 1.12 | 1.23 (0.71-2.14) | 0.86 (0.43-1.71) |
| -2 to <-1 | 27,080 | 22 | 0.81 | 0.89 (0.57-1.40) | 0.83 (0.52-1.32) |
| -1 to <1 | 150,193 | 137 | 0.91 | 1.00 | 1.00 |
| 1 to <2 | 52,929 | 49 | 0.92 | 1.02 (0.73-1.41) | 0.97 (0.70-1.36) |
| 2 to <4 | 23,249 | 34 | 1.46 | 1.60 (1.10-2.34) | 1.57 (1.06-2.32) |
| ≥4 | 2,102 | 5 | 2.38 | 2.61 (1.07-6.38) | 2.53 (0.99-6.46) |
|  |  | **Meconium aspiration** | | | |
|  |  |  |  | OR (95% CI) | |
| Weight change (BMI units) | Total | No. | Rate/1000 | Crude^a^ | Adjusted^b^ |
| <-2 | 12,445 | 3 | 0.24 | 0.45 (0.14-1.43) | 0.25 (0.06-1.12) |
| -2 to <-1 | 27,088 | 14 | 0.52 | 0.97 (0.55-1.71) | 0.93 (0.51-1.68) |
| -1 to <1 | 150,161 | 80 | 0.53 | 1.00 | 1.00 |
| 1 to <2 | 52,943 | 20 | 0.38 | 0.71 (0.43-1.16) | 0.68 (0.42-1.13) |
| 2 to <4 | 23,248 | 17 | 0.73 | 1.37 (0.81-2.32) | 1.16 (0.66-2.04) |
| ≥4 | 2,099 | 4 | 1.90 | 3.58 (1.31-9.79) | 2.11 (0.62-7.16) |
|  |  |  |  |  |  |
|  |  |  |  |  |  |

^a^Crude odds ratios are based on 263,014 second births with normal BMI in the second pregnancy, of whom 1,102 had low (0-6) Apgar score at 5 minutes. Corresponding numbers for neonatal seizures in the crude model were 268,005 and 261 and for meconium aspiration 267,984 and 138.

^b^Adjusted for BMI in first pregnancy, smoking in 2^nd^ pregnancy, maternal age at second birth, interpregnancy interval, mother’s education and country of birth, and year of 2^nd^ birth. Adjusted analyses for Apgar score 0-6 at 5 minutes are based on 258,331 second births with complete information on co-variates. Number of births with Apgar score 0-6 at 5 minutes were 1,078. Adjusted analyses for neonatal seizures and meconium aspiration syndrome are based on 263,201 and 263,180 second births with complete information on co-variates. Number of infants with neonatal seizures in the adjusted model was 256 and. meconium aspiration was 134.
