# Supplementary material for: Inter-pregnancy Weight Change and Risks of Severe Birth-Asphyxia-Related Outcomes in Singleton Infants Born at Term: A Nationwide Swedish Cohort Study
Source: PLoS Med. 2016 Jun 7;13(6):e1002033. doi: 10.1371/journal.pmed.1002033 (PMC4896455; doi:10.1371/journal.pmed.1002033)
Supplement: S3 Table — (DOCX) [file pmed.1002033.s004.docx]

**S3 Table. Maternal characteristics in women with and without data on interpregnancy weight change**

|  | Known weight change Unknown weight change | | | | | | |
| --- | --- | --- | --- | --- | --- | --- | --- |
|  | No. |  | % |  | No. |  | % |
| **Total** | 429,983 |  | 100 |  | 102,875 |  | 100 |
|  |  |  |  |  |  |  |  |
| **Maternal factors** |  |  |  |  |  |  |  |
|  |  |  |  |  |  |  |  |
| BMI  at 1^st^ pregnancy |  |  |  |  |  |  |  |
| < 18.5 | 13,222 |  | 3.08 |  | 1,245 |  | 3.21 |
| 18.5-24.9 | 298,788 |  | 69.49 |  | 27,419 |  | 70.62 |
| 25-29.9 | 88,258 |  | 20.53 |  | 7,515 |  | 19.36 |
| 30-34.9 | 22,320 |  | 5.19 |  | 1,913 |  | 4.93 |
| ≥35 | 7,395 |  | 1.72 |  | 732 |  | 1.89 |
| Data missing | - |  | - |  | 64,051 |  | 62.3 |
|  |  |  |  |  |  |  |  |
| Maternal age  at 2^nd^ delivery (years) |  |  |  |  |  |  |  |
| ≤24 | 47,872 |  | 11.13 |  | 11,060 |  | 10.75 |
| 25-29 | 142,298 |  | 33.09 |  | 31,311 |  | 30.44 |
| 30-34 | 166,944 |  | 38.83 |  | 40,363 |  | 39.23 |
| ≥35 | 72,869 |  | 16.95 |  | 20,141 |  | 19.58 |
|  |  |  |  |  |  |  |  |
| Smoking in 2^nd^ pregnancy |  |  |  |  |  |  |  |
| No | 391,880 |  | 92.10 |  | 77,519 |  | 91.27 |
| Yes | 33,636 |  | 7.90 |  | 7,412 |  | 8.73 |
| Data missing | 4,467 |  | 1.04 |  | 17,944 |  | 1.73 |
|  |  |  |  |  |  |  |  |
| Interpregnancy interval (years) |  |  |  |  |  |  |  |
| <1 | 75,805 |  | 17.63 |  | 18,848 |  | 18.32 |
| 1 to <3 | 258,356 |  | 60.10 |  | 59,958 |  | 58.28 |
| 3 to <5 | 63,255 |  | 14.71 |  | 15,311 |  | 14.88 |
| ≥5 | 32,567 |  | 7.57 |  | 8,758 |  | 8.51 |
|  |  |  |  |  |  |  |  |
| Education (years) |  |  |  |  |  |  |  |
| ≤11 | 98,086 |  | 22.99 |  | 24,496 |  | 24.10 |
| 12-14 | 178,495 |  | 41.83 |  | 40,327 |  | 39.67 |
| ≥15 | 150,116 |  | 35.18 |  | 36,829 |  | 36.23 |
| Data missing | 3,183 |  | 7.40 |  | 1,223 |  | 11.88 |
|  |  |  |  |  |  |  |  |
| Mother’s country  of birth |  |  |  |  |  |  |  |
| Nordic | 372,940 |  | 86.73 |  | 87,980 |  | 85.52 |
| Non-Nordic | 57,043 |  | 13.27 |  | 14,895 |  | 14.48 |
|  |  |  |  |  |  |  |  |
| Chronic hypertension |  |  |  |  |  |  |  |
| No | 427,880 |  | 99.51 |  | 102,369 |  | 99.51 |
| Yes | 2,103 |  | 0.49 |  | 506 |  | 0.49 |
|  |  |  |  |  |  |  |  |
| Preeclampsia |  |  |  |  |  |  |  |
| No | 425,009 |  | 98.84 |  | 101,693 |  | 98.85 |
| Yes | 4,974 |  | 1.16 |  | 1,182 |  | 1.15 |
|  |  |  |  |  |  |  |  |
| Diabetes |  |  |  |  |  |  |  |
| No | 425,271 |  | 98.90 |  | 101,724 |  | 98.88 |
| Gestational diabetes | 3,322 |  | 0.77 |  | 757 |  | 0.74 |
| Pregestational diabetes | 1,390 |  | 0.32 |  | 394 |  | 0.38 |
|  |  |  |  |  |  |  |  |
| **Neonatal outcomes** |  |  |  |  |  |  |  |
| Apgar 0-6 | 2,246 |  | 0.53 |  | 578 |  | 0.57 |
| Neonatal seizures | 538 |  | 0.13 |  | 120 |  | 0.12 |
| Meconium aspiration | 294 |  | 0.07 |  | 78 |  | 0.08 |
|  |  |  |  |  |  |  |  |
|  |  |  |  |  |  |  |  |
