# Supplementary material for: Inter-pregnancy Weight Change and Risks of Severe Birth-Asphyxia-Related Outcomes in Singleton Infants Born at Term: A Nationwide Swedish Cohort Study
Source: PLoS Med. 2016 Jun 7;13(6):e1002033. doi: 10.1371/journal.pmed.1002033 (PMC4896455; doi:10.1371/journal.pmed.1002033)
Supplement: S1 Text — (DOCX) [file pmed.1002033.s005.docx]

# Analysis plan

“Risk of birth asphyxia and related outcomes in relation to maternal interpregnancy weight change”

## Investigators

Martina Persson, Stefan Johansson, Sven Cnattingius

## Ethics approval

Ethic approval for this study was obtained from the Research Ethics Committee at Karolinska Institutet in Stockholm, Sweden (No 2012/4:9).

## Hypothesis

We have previously demonstrated that risks of birth asphyxia and related outcomes (meconium aspiration and neonatal seizures) increase with increasing maternal BMI. In this study our pre-specified hypothesis was that risks of birth asphyxia and related outcomes would increase with weight gain between consecutive pregnancies.

## Study base

All mothers mothers with first and second live singleton term births (≥37 completed weeks) in the Medical Birth Register (n= 533,535) between 1992 and 2012.

## Study population

Infants with data on Apgar scores at 1 and 5 minutes. Data on maternal country of birth and information on interpregnancy interval are required.

## Exposure

Weight gain between pregnancies expressed as BMI units and categorized as less than 2 units -2 to less than -1, -1 to less than 1,1 to less than 2, 2 to less than 4, and 4 or more units.

Obesity related disorders: pregestational diabetes (type 1 diabetes and type 2 diabetes), gestational diabetes, chronic hypertension, preeclampsia.

## Covariates

Maternal age at 2:nd delivery, BMI at first delivery, height, interpregnancy interval, education, smoking habits 2:nd pregnancy, maternal country of birth.

## Outcomes

Proxy for birth asphyxia: Apgar score < 6 at 5 min. Meconium aspiration, neonatal seizures (ICD codes).

## Statistical analyses

Maternal characteristics: distribution of co-variates in mothers to offspring with birth asphyxia and related outcomes.

Calculation of rates of all outcomes, crude and multivariate logistic regression analyses for all outcomes.

Stratification by maternal first trimester BMI (i.e. overweight yes/no)

Sensitivity analyses: repeated analyses as above but with women with offspring to mothers with obesity related disorders excluded.

This design and planned analyses described above were decided beforehand.

After these first analyses were performed, we wanted to explore more carefully the following:

1. Risks of birth asphyxia and related outcomes in relation to interpregnancy weight gain in offspring of mothers who were normal weight in the second pregnancy (S2 Table)
2. Distribution of maternal characteristics in pregnancies to mothers with and without data on interpregnancy weight change (S3 Table)
